# Comparison of three different dressings for partial thickness burns in children: study protocol for a randomised controlled trial

**DOI:** 10.1186/1745-6215-14-403

**Published:** 2013-11-25

**Authors:** Emma Gee Kee, Roy M Kimble, Leila Cuttle, Kellie Stockton

**Affiliations:** 1 Centre for Children’s Burns and Trauma Research, Queensland Children’s Medical Research Institute, University of Queensland, Royal Children’s Hospital, Brisbane, QLD, Australia; 2 Children’s Burns and Trauma Research, Queensland Children’s Medical Research Institute, Queensland University of Technology, Brisbane, QLD, Australia

**Keywords:** Child, Burn injuries, Partial thickness, Silver dressings, Healing, Pain, Distress, Randomised clinical trial

## Abstract

**Background:**

In the paediatric population, pain and distress associated with burn injuries during wound care procedures remain a constant challenge. Although silver dressings are the gold standard for burn care in Australasia, very few high-level trials have been conducted that compare silver dressings to determine which will provide the best level of care clinically. Therefore, for paediatric patients in particular, identifying silver dressings that are associated with lower levels of pain and rapid wound re-epithelialisation is imperative. This study will determine whether there is a difference in time to re-epithelialisation and pain and distress experienced during wound care procedures among Acticoat™, Acticoat™ combined with Mepitel™ and Mepilex Ag™ dressings for acute, paediatric partial thickness burns.

**Methods/Design:**

Children aged 0 to 15 years with an acute partial thickness (superficial partial to deep partial thickness inclusive) burn injury and a burn total body surface area of ≤10% will be eligible for the trial. Patients will be randomised to one of the three dressing groups: (1) Acticoat™ or (2) Acticoat™ combined with Mepitel™ or (3) Mepilex Ag™. A minimum of 28 participants will be recruited for each treatment group. Primary measures of pain, distress and healing will be repeated at each dressing change until complete wound re-epithelialisation occurs or skin grafting is required. Additional data collected will include infection status at each dressing change, physical function, scar outcome and scar management requirements, cost effectiveness of each dressing and staff perspectives of the dressings.

**Discussion:**

The results of this study will determine the effects of three commonly used silver and silicone burn dressing combinations on the rate of wound re-epithelialisation and pain experienced during dressing procedures in acute, paediatric partial thickness burn injuries.

**Trial registration:**

Australian New Zealand Clinical Trials Registry ACTRN12613000105741

## Background

The ultimate goal of burn wound healing is to promote early closure as this has considerable influence on the long-term quality and appearance of a hypertrophic scar. It has been demonstrated that even scars considered small in size can contribute to negative psychosocial outcomes for children, hence the importance of effective wound healing techniques [1,2]. The relationship between scar formation and time taken to re-epithelialise in children is well understood by burn clinicians. According to Cubison and colleagues [3], partial thickness burns that re-epithelialise within the optimal time period of 10–14 days generally do so without scarring, and those taking more than 3 weeks will invariably scar. Burns re-epithelialising at between 2 and 3 weeks will have variable amounts of scar tissue laid down depending on many factors including skin type, anatomical location of the burn and age of the child [3].

Children whose burn wounds re-epithelialise at more than 3 weeks post-injury are at a high risk of residual or hypertrophic scarring [3]. Scar management therapy facilitated by occupational therapists to manage and prevent hypertrophic scarring currently involves the use of various types of silicone contact media, pressure garments and splints. Engagement in scar management therapy typically occurs for 18 months or until scars reach maturity. While these scar management techniques are successful for some children in managing scars, there are inevitably children who require ongoing treatment of their scar tissue as they grow older. Scars generally do not grow with the child, and if situated around a joint, can lead to joint contracture and loss of function, resulting in ongoing scar reconstruction to keep up with the child’s growing body [4]. Therefore the initial care of the burn wound and choice of burn dressing are vital in creating the ideal healing environment to ensure rapid re-epithelialisation of the wound and to avoid the possibility of hypertrophic scarring.

In the past 30–40 years, children with burns were treated with daily baths, dressing changes and antiseptic or topical silver sulfadiazine-based creams; however even with appropriate pain relief these procedures were often very distressing and painful for children [5]. It has been well documented that burn wound care procedures are highly traumatic for children and the resultant stress has been shown to interrupt and delay the cascade of wound healing [6]. Decreasing the pain and distress experienced during a dressing change procedure can have positive implications psychosocially for the child as well as encouraging re-epithelialisation of the wound within the optimal healing timeframe [7]. Therefore in the paediatric population, careful attention also needs to be made concerning choosing a dressing that can be applied and removed with minimal pain and stress to the child. A dressing that requires infrequent reapplication also has obvious benefits by decreasing the number of dressing change procedures the child has to undergo.

Small to medium-sized partial thickness burns are mainly managed in the outpatient setting using specialised dressings that promote moist wound healing and prevent wound infection [8]. Unfortunately the antibacterial agents in burn dressings are also cytotoxic to keratinocytes; therefore a fine balance is required between the prevention of a wound infection and the promotion of wound healing [9]. Despite the known cytotoxic effects of antibacterial dressings, they help prevent not only infections (which can delay the rate of re-epithelialisation) but also the possibility of toxic shock syndrome (TSS), which is most likely to occur in the first 3 days post-burn injury and if left untreated can be fatal [10-12].

The standard of care in burn dressings for small to medium partial thickness burns has changed in the last 10–15 years. Currently silver-depositing fabric and foam dressings are the gold standard used to manage the bio-burden of a wound, with or without a silicone skin interface [13]. Acticoat™ (Smith & Nephew, Hull, UK) has been available since the 1990s and is the most widely used silver dressing in the developed world. It needs to be moistened with sterile water to promote the release of silver onto the burn wound and can be left on for 3–4 days. Acticoat™ may be used alone or in combination with a Mepitel™ dressing (Mölnlycke Healthcare, Mikkeli, Finland), which acts as a non-stick interface between Acticoat™ and the burn wound. Mepitel™ is a silicone-coated nylon grid product that utilises non-stick Safetac™ technology to reduce the pain and trauma experienced by children during dressing changes and the amount of silver in direct contact with the wound. Mepilex Ag™ (Mölnlycke Healthcare, Mikkeli, Finland) has been available since 2007 and is a soft foam, silver-impregnated dressing that absorbs exudate and maintains a moist wound environment [14]. Silver particles are released from Mepilex Ag™ when it is moistened from direct contact with wound exudate. It also has a silicone Safetac™ interface layer incorporated within its design that, in a similar way to the Mepitel™, serves to promote easy removal of the dressing and reduce pain and trauma during dressing changes [14].

Many trials have been conducted regarding the efficacy of silver dressings for treating burn injuries, using topical silver sulfadiazine applications as the control or comparator dressing. However, these silver sulfadiazine applications have been shown to delay re-epithelialisation and are painful to apply and remove, indicating the possibility of bias in the results of these trials [13,15,16]. Additionally, despite the large number of silver-impregnated burn dressings that have become available on the market, very few high-level trials have been conducted that compare these dressings in paediatric or adult patients. For paediatric patients in particular, identifying silver dressings that are associated with lower levels of pain, require fewer reapplications and promote a fast rate of re-epithelialisation is vital.

The aim of this study is to then determine whether one of three silver- and silicone-containing burn dressings—Acticoat™, Acticoat™ combined with Mepitel™ or Mepilex Ag™—will be more effective in terms of pain experienced and the rate of re-epithelialisation of acute, partial thickness burns in children.

The following protocol for this study has been reported as per CONSORT guidelines [17].

## Methods/Design

### Ethics approval

This study is registered with the Australian New Zealand Clinical Trials Registry (ACTRN12613000105741) and approved by the Queensland Children’s Health Services (Royal Children’s Hospital) Human Research Ethics Committee and The University of Queensland Ethics Committee.

### Study design

**Figure 1:**
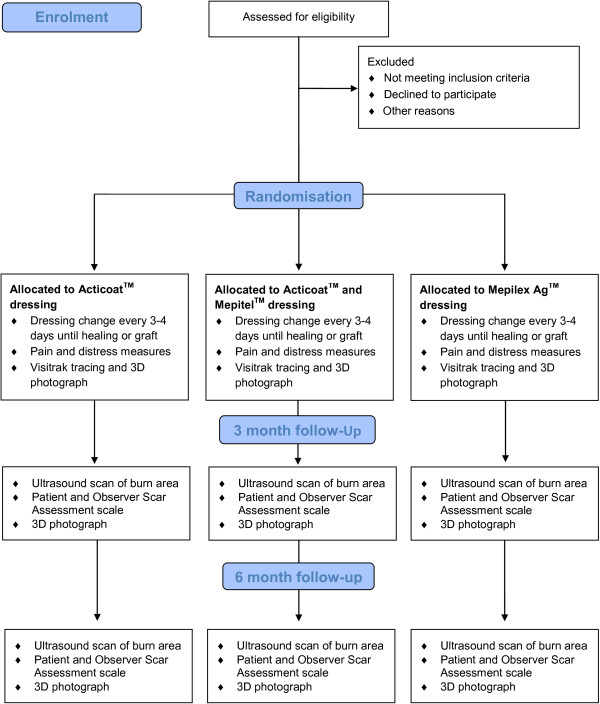
**Data collection flowchart.** The chronological timing and order of data collection within the burn wound care procedure framework.

This study is a prospective, randomised controlled trial. Participants will be randomised to receive one of three commonly used dressings for burn wounds, (1) Acticoat™, (2) Acticoat™ and Mepitel™ combined or (3) Mepilex Ag™, in order to determine the effects of the dressings on pain and the rate of re-epithelialisation in acute, partial thickness burns in children. It is hypothesised that silver dressings with a silicone interface, compared to no silicone interface, will decrease the time to re-epithelialisation of a burn injury and decrease the amount of pain and distress experienced during dressing changes within a paediatric population. The design of data collection is displayed in Figure 1.

### Study setting

Participants for this study will be recruited from the Royal Children’s Hospital, Brisbane, Australia. Children presenting to the Department of Emergency (DEM) from March 2013 will be screened on presentation by a surgical registrar for eligibility to this study.

### Participants

#### Inclusion criteria

Children who are aged 0 to 15 years with an acute partial thickness (superficial partial to deep partial thickness inclusive) burn injury and a burn total body surface area of ≤10%, presenting at the DEM within the first 72 h post-injury will be considered for inclusion in this study.

#### Exclusion criteria

Children will be excluded from this study if they present >72 h post burn; have received silver dressings prior to presentation at the Royal Children’s Hospital; have sustained a superficial (erythema only) or full thickness burn; have sustained a chemical or friction burn; present with cold, flu or viral symptoms (e.g. upper respiratory tract infection); have received inappropriate first aid (e.g. dirty water); have a known reaction to silver products; are non-English speaking; have a cognitive impairment; or are currently involved with Department of Communities (Child Safety). Additionally, any children who have burn wounds requiring grafting, contract an infection or need to change dressing type during the course of their treatment will only have data collected up until that point in time, as these changes in clinical care will void the intervention they have been randomised into for the study. These exclusions will not be known until after the child is recruited and randomised because of the variability of patient and wound management needs.

Apart from the dressings received, children participating in this study will receive the same standard medical care as those children not participating in the study.

### Recruitment

A surgical registrar will be notified of a patient with a burn injury by DEM staff and will then determine the patient’s eligibility according to study criteria. The potential participants and their parents/caregivers will be notified of their eligibility to participate in this study by the registrar. Parents/caregivers will then be asked if a member of the research team can speak to them about the study. Informed caregiver consent and child assent (for children 6 years and above) will be gained by an investigator on the study after a discussion with the child and their caregiver regarding the information in the Patient Information Sheet. Once consent has been gained, the investigator will randomise the patient to one of the three dressing treatment arms. Randomisation will be completed using a computerised random number generator and randomisations will be enclosed in sealed, opaque envelopes by an independent party. All dressings will be carried out by staff experienced in burn dressing application. Baseline measures (pain and behavioural scores from child, nurse and parent, pulse rate and respiratory rate) will be recorded by the primary investigator prior to and after the dressing has been applied. The patient will then present at our burn centre every 3–4 days for subsequent dressing changes. Any analgesia given to the patient will be recorded.

The first 3 days post-burn injury often have the highest risk of toxic shock syndrome, which can be potentially fatal if left untreated [10,18]. While the incidence rate of toxic shock syndrome at our burn centre is extremely low to non-existent, phone calls will be made to each participant by the primary investigator within this period. A set of standardised questions will be used to screen for any toxic shock syndrome symptoms and to assess how the child and their parents or caregivers are managing the dressing, and all responses will be recorded.

### Intervention

The intervention will be one of the Acticoat™, Acticoat™ combined with Mepitel™ or Mepilex Ag™ dressing arms that the participant is randomised into on presentation. This dressing will then be used to treat the burn wound and will be replaced every 3–4 days at each dressing change until re-epithelialisation occurs or grafting is required. The dressings will be applied according to the manufacturer’s instructions.

### Data collection

Demographic information and clinical details will be obtained from the caregiver and the patient’s chart regarding the mechanism of the burn injury, site of the injury, total body surface area of the burn and first aid the patient received. Children over the age of 4 years will be given the Ditto™ (virtual reality) device (Diversionary Therapy Technologies, Queensland, Australia) to engage in the “Bobby got a Burn” procedural preparation story at their first dressing change. These children may also engage in a choice of games or stories on the Ditto™ during all dressing changes. At the first dressing change only, each participant will have their burn/s scanned using the LDI2-BI (VR) Laser Doppler Imager (LDI) (Moor Instruments, Axminster, Devon, UK), which measures wound depths (in blood perfusion units) as a percentage of total wound area. The laser Doppler imager scan will provide an objective assessment of burn depth to enable a comparison of treatment group demographics [19]. Participants who have burn wounds on different body parts will have separate scans completed for each body part involved. If burn wounds are circumferential or across curved areas, more than one scan will be taken at the frontal, lateral and medial aspects to obtain an accurate scan. In addition, blinded photo reviews of burn depth will be undertaken by a panel of burn specialists (consultants and nurses).

At each dressing change appointment, pain, behaviour and physiological measures (pulse rate and respiratory rate) will be taken before and after dressing removal and before and after the re-application of a new dressing. The time taken for the dressing removal, cleaning and re-application procedure, the number of nurses required to complete the entire procedure, quantity of dressings used and analgesia given to the participant will be recorded. A tracing of the wound using Visitrak™ grids (Smith & Nephew, Hull, UK) and a photograph using the 3D LifeViz Camera (Quantificare, Cedex, France) will be taken. Nursing staff treating the child will be surveyed on a 5-point Likert scale regarding the ease of use of the dressing for that particular child. Parents or caregivers and children over the age of 8 years will be surveyed on a 5-point Likert scale regarding their physical function while wearing the dressing.

All participants in the study will have their dressings changed every 3–4 days at our burn centre as a standard measure. This protocol will be followed until full re-epithelialisation of the wound occurs or grafting of the wound is required [20]. After full re-epithelialisation occurs, participants will be followed up at 3 months and then 6 months to assess skin appearance and/or the presence of scarring.

### Primary outcome measures

#### Days to re-epithelialisation

The number of days from the burn injury date until wound re-epithelialisation occurs, surface area of the wound and percentage of wound re-epithelialisation will be calculated by four methods: (1) clinical judgement from the consultant; (2) use of Visitrak™ grids; (3) 3D camera photographs and analysis on specialist computer software; and (4) blinded review of the 3D photographs by a panel of burn specialists (consultants and nurses).

The 3D photographs and Visitrak™ tracings will be taken of a participant’s burn wound at every dressing change. A ruler will be included in all 3D photographs for measurement calibration in the associated software package Dermapix™ (Quantificare, Cedex, France). The 3D photographs will be analysed by the primary investigator on the Dermapix™ software programme to calculate the burn wound surface area and area of re-epithelialisation of the wound from each dressing change. The Visitrak™ will also be used to trace around total burn area and re-epithelialised areas of the burn wounds at each dressing change in order to calculate wound surface area and percentage of re-epithelialisation.

A blinded review of the 3D photographs will be undertaken by a panel of burn wound specialists (consultants and nursing staff) who will assess the progress of re-epithelialisation and appearance of each participant’s burn wound after data collection has been completed.

#### Pain

Pain and distress will assessed by obtaining: (1) the participant’s self-report of pain intensity using the Faces Pain Scale-Revised (FPS-R) (if participant is aged 3 years or over) [21]; (2) the parent’s report of the participant’s pain intensity using a Visual Analogue Scale-Pain (VAS-P) (alternatively, if participants are aged 8 years or over they will complete the VAS-P in lieu of the caregiver) [22]; (3) the nurse’s observational rating of the participant’s pain and distress using the Face, Legs, Activity, Cry, Consolability (FLACC) scale [23]; and (4) pulse and respiratory rates of the participant.. All four of these measures will be taken before and after dressing removal and before and after dressing application. Pulse and respiratory rates will be recorded once at each time point to provide an objective measure of pain and distress in participants, as increases in these physiological measures have been shown to be indicative of pain and distress [6,24] Any analgesic and/or sedative medication administered to the participant at each dressing change will also be recorded.

Face scales are particularly useful for obtaining self-report pain scores from younger children as they have been reported to be easier to follow than other types of self-report scales and are well-validated measures [25]. The FPS-R is clinically valid for a paediatric population and was chosen over other faces pain scales because of its psychometrically sound characteristics including the exclusion of smiling faces or tears, thus eliminating the possible confusion of pain intensity and affect by children [21]. The VAS-P is a valid and reliable self-report measure of acute pain in children over the age of 8 years [22]. The VAS-P is not valid for children under the age of 8, but is valid as a parent-report measure of pain and therefore parents and caregivers of these children in this study will instead score their child’s pain level [22]. The FLACC scale is a valid and reliable behavioural observer-report (nurse or health professional) measure of a child’s pain. Self-reports of pain intensity can be difficult to ascertain and inaccurate when a child is receiving sedative and analgesic medications or is too young to verbalise their pain level; therefore the use of the FLACC scale can be a suitable and accurate alternative [23].

### Secondary outcome measures

#### Physical function

A self-report of the participants’ ease of movement while wearing the dressing will be obtained using a 5-point Likert scale question from children over the age of 8 years or their caregiver at the first dressing change [26].

#### Grafting

A note will be recorded if a participant’s wound requires grafting. Data collection will cease on that date for the participant as different dressings are used for wounds that are grafted; however all data preceding that point will be included for analysis.

#### Infection

At each dressing change, the wound will be assessed clinically by the consultant. If the consultant deems an infection to be present, a swab will be taken from the wound for confirmation and identification.

#### Cost-effectiveness of dressings

At each dressing change, nursing time to apply the dressing, the amount and size of each dressing used, and any other resources required will be recorded and a cost-analysis will then be calculated. Additionally, dressings required for scar management will also be recorded and analysed.

#### Scar assessment

If further scar management treatment is required after full re-epithelialisation of the burn injury has occurred, a referral will be made by the treating consultant to the scar management clinic. At 3 months following full re-epithelialisation of the burn injury, a face-to-face follow-up will be completed with all participants to conduct a skin and/or scar review in conjunction with Occupational Therapy. An ultrasound scan (BT12 Venue 40 MSK, GE Healthcare) [19] will be taken of the burn area to measure the height of the scar, and digital and 3D photographs will also be taken. The Patient and Observer Scar Assessment Scale will also be completed with the child (if over the age of 6 years) and/or caregiver. All scar management resources used with the participant will be recorded for a cost-analysis. At 6 months post-full re-epithelialisation, the same measures will be repeated.

#### Burn centre staff perspectives on dressings

Nursing staff, consultants and occupational therapists from our burn centre will be surveyed regarding their opinions of the three dressings prior to the commencement of data collection. The survey questions will be standardised and consist of Likert scales, tick boxes with a selection of responses and additional space for comments regarding the dressings. Nursing staff will be surveyed again using the same questions after data collection has ceased to examine any changes in their perceptions of the dressings after using all three for an extended period of time.

### Blinding

The dressing used for each participant cannot be completely masked, as the Acticoat™ dressing stains the healthy skin around a burn wound brown. The expert panel of burn wound specialists will be blinded where possible; however the primary investigator and expert panel may still be able to deduce what dressing a participant received. The primary investigator will also be present when dressings are being applied and removed to obtain pain scores from the participant, caregiver and nursing staff, and will see which dressing is being used on the child. Wherever possible during data collation and analysis, treatment groups will be de-identified.

### Discontinuation/adverse events

The infection of the burn wound is a potential adverse effect of any of the burn dressings. Infection rates at our burn centre are extremely low; however if any adverse effects such as infection occur, participants will only have data collected up until that point in time analysed, as clinical care (including dressing type) may change to address the adverse event. Additionally, if a consultant feels that a particular dressing is not appropriate for a participant’s care, they may change to a different dressing. If this occurs, data collection for this participant will cease from this date. All data preceding this date will be included for analysis.

### Statistics

#### Sample size

The primary outcome measure will be days until wound re-epithelialisation. Previous data in paediatric burn patients demonstrated re-epithelialisation within 15 (SD = 4) days and a minimally clinically important difference is 3 days [6]. Thus sample size was calculated at 28 per group at 80% power with an α of 0.05. Allowing for 20% loss to follow-up, a total of 100 participants will be required. The burn centre treats 800 children per year with burns so recruitment for this study should be achievable within 1 year. This sample size will also be adequate to find a significant difference in data collected from pain scores, powered on data from a previous study [6].

#### Data analysis

All statistical analyses will be conducted using SPSS 21 (IBM Corp., Armonk, NY, USA). Generalised linear models, estimating variance appropriately for repeated measures, will be used to determine whether there are differences among the Acticoat™ group, the Acticoat™ combined with Mepitel™ group and the Mepilex Ag™ group in primary and secondary outcomes. Changes in the intervention effects will be examined with burn depth, burn total body surface area, mechanism of injury, anatomical location of the burn, skin type, ease of dressing removal and application and physical function considered a priori to be of potential interest. All data will be analysed as intention to treat and on a per protocol basis, with the intention to treat analysis being the primary approach for this trial. Any missing data will be handled using the multiple imputation method. All tests will be two-tailed and only those with a *p* value <0.05 will be considered statistically significant.

### Data storage

Data will be securely stored in a locked filing cabinet in a secure area of Queensland Children’s Medical Research Institute, The University of Queensland. Data will be entered into an SPSS spreadsheet and any incomplete data will be coded as missing, unknown or not applicable. The full data set will be cleaned and checked before being locked for analysis. On completion of the study, data will be kept for a period of 15 years in accordance with NHMRC guidelines.

## Discussion

Pain and distress during dressing change procedures remain a major challenge when treating acute paediatric burn injuries and can have a negative effect on healing [6]. Although silver dressings are the gold standard for burn care in Australasia, very few high-level trials have been completed comparing the clinical utilities of these dressings, particularly in relation to pain and rates of re-epithelialisation in paediatric burn patients. Additionally, the majority of trials that are available on these dressings only show the benefits of using these silver dressings in comparison with silver sulfadiazine creams for burn injuries and are not specific to paediatric or adult patients [15]. Therefore, for paediatric patients in particular, identifying silver dressings that are associated with lower levels of pain and rapid wound re-epithelialisation is imperative not only for clinical care, but also to facilitate further evidence based practice in this field.

A clear link has been established between the rate of re-epithelialisation of a burn wound and the risk of hypertrophic scarring. Partial thickness burn injuries that heal within 10–14 days are at a very low risk of developing hypertrophic scarring, whereas wounds that re-epithelialise within 2 to 3 weeks will inevitably produce some scar tissue, and those wounds taking more than 3 weeks to re-epithelialise are likely to result in hypertrophic scarring [3]. Given this knowledge, if there is a silver dressing that is associated with a reduced time for a burn injury to re-epithelialise, there is a possibility that partial thickness burns can be healed within the optimal time period of 10–14 days, which could subsequently reduce the risk of hypertrophic scarring and the need for reconstructive surgery in paediatric patients with this depth of burn.

The associated pain experienced during dressing removal and re-application by paediatric patients is a significant problem for health professionals. Previous studies have shown that an increase in pain, anxiety and distress during various types of wound care procedures can have a negative effect on the healing cascade [6,7,24]. This negative effect on the rate of re-epithelialisation in paediatric patients with burn injuries may increase the likelihood of hypertrophic scarring as a result of the injuries not re-epithelialising in the optimal time period. It is therefore imperative to identify a silver dressing that promotes a fast rate of re-epithelialisation and that induces the least amount of pain and discomfort on removal and application for paediatric patients. Dressings that are comfortable and easy to move in and that require infrequent changes are also beneficial for the paediatric population.

While every precaution possible will be taken for this study, there still may be some limitations involved with data collection. All children recruited into the study will have a laser Doppler imager scan taken of their burn wound [19]. Although the scan can be completed for children of any age, children are required to remain still for a period of time with their wound exposed to the air and free of dressings while the scan is being taken. Given that this study is recruiting all children under the age of 16 years, completing these scans may be difficult if not impossible in some circumstances because of the age of the child and associated pain from the exposed wound. Thus, to compensate for this limitation, a blinded photo review of burn depth by a panel of burn specialists will also be undertaken. Additionally, completing Visitrak™ tracings on some children may be a challenge, as they are again required to remain still and may experience pain or discomfort when the Visitrak™ grid is in direct contact with their wound.

### Significance of study

In Australasia, the standard of care in burn dressings for small to medium partial thickness burns has changed over the past 10–15 years. Currently, silver-containing dressings are used as the gold standard of burn care to prevent infection and promote healing. However, despite the large number of silver burn dressings available on the market, very few high level trials have been conducted in paediatric or adult patients. This study aims to determine the effect of Acticoat™, Acticoat™ combined with Mepitel™ and Mepilex Ag™ burn dressings on the rate of re-epithelialisation of partial thickness burn wounds and pain and distress levels during the treatment of these injuries in the paediatric population.

## Trial status

This study commenced recruitment on 18 March 2013. Completion of participant recruitment is planned by December 2013 and data collection is likely to continue to June 2014 (with data collection continuing until 6 months post re-epithelialisation of participant’s burns).

## Abbreviations

DEM: Department of Emergency; FLACC: Face, Legs, Activity, Cry, Consolability; FPS-R: Faces pain scale-revised; RCH: Royal Children’s Hospital; VAS-P: Visual Analogue scale-pain.

## Competing interests

This clinical trial is partially financially supported by a grant given to the University of Queensland by Mölnlycke Healthcare. Despite this financial support, Mölnlycke Healthcare had no part in the study design and data collection of this project, nor will they have any involvement in the analysis or publication of results. The principal researcher has no financial interest in the Acticoat™, Mepitel™ or Mepilex Ag™ dressings or the Mölnlycke Healthcare Company and is a student of the University of Queensland.

## Authors’ contributions

EGK, RMK, LC and KS all made substantial contributions to the design of this trial. EGK wrote the draft manuscript with substantial input from KS. All authors provided critical review of the article and approved the final manuscript.
